# Effects of the 5-m Shuttle Run Test on Markers of Muscle Damage, Inflammation, and Fatigue in Healthy Male Athletes

**DOI:** 10.3390/ijerph17124375

**Published:** 2020-06-18

**Authors:** Omar Boukhris, Khaled Trabelsi, Raouf Abdessalem, Hsen Hsouna, Achraf Ammar, Jordan M. Glenn, Nick Bott, Khadijah Irandoust, Morteza Taheri, Mouna Turki, Fatma Ayadi, Nicola L. Bragazzi, Florian A. Engel, Hamdi Chtourou

**Affiliations:** 1 Activité Physique, Sport et Santé, UR18JS01, Observatoire National du Sport, Tunis 1003, Tunisia; omarboukhris24@yahoo.com (O.B.); raoufabdesalem18@gmail.com (R.A.); hsen.hsouna92@gmail.com (H.H.); h_chtourou@yahoo.fr (H.C.); 2 Institut Supérieur du Sport et de l’Education Physique de Sfax, Université de Sfax, Sfax 3000, Tunisia; trabelsikhaled@gmail.com; 3 High Institute of Sport and Physical Education of Sfax, University of Sfax, Sfax 3000, Tunisia; 4 Institute of Sport Science, Otto-von-Guericke University Magdeburg, 39106 Magdeburg, Germany; ammar.achraf@ymail.com; 5 Exercise Science Research Center, Department of Health, Human Performance and Recreation, University of Arkansas, Fayetteville, AR 72701, USA; jordan@neurotrack.com; 6 Neurotrack Technologies, 399 Bradford St, Redwood City, CA 94063, USA; nick@neurotrack.com; 7 Clinical Excellence Research Center, Department of Medicine, Stanford University School of Medicine, Stanford, CA 94305, USA; 8 Department of Sport Sciences, Faculty of Social Sciences, Imam Khomeini International University, Qazvin 34148-96818, Iran; irandoust@soc.ikiu.ac.ir (K.I.); taheri_morteza@yahoo.com (M.T.); 9 Laboratoire de Biochimie, Centre Hospitalo-universitaire Habib Bourguiba, avenue El-Ferdaous, Sfax 3029, Tunisia; mouna.turki@gmail.com (M.T.); fatma.makni@rns.tn (F.A.); 10 Unité de Recherche Bases Moléculaires de la Pathologie Humaine, Faculté de Médecine de Sfax, Sfax 3029, Tunisia; 11 Department of Health Sciences (DISSAL), Postgraduate School of Public Health, University of Genoa, 16132 Genoa, Italy; 12 Institute of Sport and Sport Science, Department of Movement and Training Science, Heidelberg University, 69120 Heidelberg, Germany; florian.engel@issw.uni-heidelberg.de; 13 Department of Sport Science, Bundeswehr University Munich, 85577 Neubiberg, Germany

**Keywords:** repeated sprint, muscle damage, inflammation, fatigue, muscle soreness

## Abstract

Physical exercise is often associated with increases in muscle damage markers and inflammation. However, biomarkers of muscle damage and inflammation responses to the 5-m shuttle run test (5mSRT) have not yet been evaluated. The aim of the present study was to investigate effects of the 5mSRT on muscle damage markers, inflammation, and perception of fatigue and recovery in healthy male athletes. Fifteen male amateur team sports players (age: 20 ± 3 yrs, height: 173 ± 7 cm, body-mass: 67 ± 7 kg) participated in this study. Blood biomarkers were collected at rest, 5 min after, and 72 h after the 5mSRT to measure muscle damage (i.e., creatinine kinase (CK), lactate dehydrogenase (LDH), aspartate aminotransferase (ASAT), and alanine aminotransferase (ALAT)) and inflammation (i.e., C-reactive protein (CRP)). Best distance (BD), total distance (TD), fatigue index (FI), and percentage decrement (PD) during the 5mSRT were assessed. Perceived recovery (PRS) and delayed onset muscle soreness (DOMS) were recorded before, 5 min after, and 72 h after the 5mSRT; perceived exertion (RPE) was recorded before, during, and 72 h after the 5mSRT. Muscle damage biomarkers post 5mSRT showed a significant increase compared to pre 5mSRT (*p* < 0.001) levels ((i.e., CK (190.6 ± 109.1 IU/L vs. 234.6 ± 113.7 IU/L), LDH (163.6 ± 35.1 IU/L vs. 209.9 ± 50.8 IU/L), ASAT (18.0 ± 4.4 IU/L vs. 21.7 ± 6.2 IU/L), and ALAT (10.2 ± 3.4 IU/L vs. 12.7 ± 3.8 IU/L)) and 72 h post 5mSRT (*p* < 0.001) levels ((CK (125.3 ± 80.5 IU/L vs. 234.6 ± 113.7 IU/L), LDH (143.9 ± 36.6 IU/L vs. 209.9 ± 50.8 IU/L), ASAT (15.0 ± 4.7 IU/L vs. 21.7 ± 6.2 IU/L), and ALAT (8.6 ± 2.4 IU/L vs. 12.7 ± 3.8 IU/L)). CRP was also significantly higher post 5mSRT compared to pre 5mSRT (2.1 ± 2.5 mg/L vs. 2.8 ± 3.3 mg/L, *p* < 0.001) and 72 h post 5mSRT (1.4 ± 2.3 mg/L vs. 2.8 ± 3.3 mg/L, *p* < 0.001). Significant correlations were reported between (i) physical performance parameters (i.e., PD, FI, TD, and BD), and (ii) markers of muscle damage (i.e., CK, LDH, ASAT, and ALAT) and inflammation (i.e., CRP). Similarly, DOMS and RPE scores were significantly higher post 5mSRT compared to pre 5mSRT (2.4 ± 1.0UA vs. 6.7 ± 1.1UA and 2.1 ± 0.6 UA vs. 8.1 ± 0.6 UA, respectively *p* < 0.001) and 72 h post 5mSRT (1.9 ± 0.7 UA vs. 6.7 ± 1.1 UA and 1.5 ± 0.6 UA vs. 8.1 ± 0.6 UA, respectively *p* < 0.001). PRS scores were significantly lower post 5mSRT as compared to pre 5mSRT (6 ± 1 UA vs. 3 ± 1 UA, *p* < 0.001) and 72 h post 5mSRT (7 ± 1 UA vs. 3 ± 1 UA, *p* < 0.001). Significant correlations existed between (i) performance parameters (PD, FI, TD, and BD) and (ii) RPE, PRS, and DOMS. The 5mSRT increased biomarkers of muscle damage and inflammation, as well as the DOMS and RPE and reduced the PRS. Seventy-two hours was sufficient for fatigue recovery induced by the 5mSRT. PD is better than FI for the calculation of performance decrements during the 5mSRT to represent fatigue.

## 1. Introduction

Repeated short-duration maximal sprints, interspersed with brief recovery periods, are common during many team sports such as soccer, rugby, and handball [1]. As a result, repeated sprint exercises represent an important component of team-sport athletics and are used as a training method to improve performance [2]. Characterized by intermittent, short duration, and high intensity activity bouts, the 5-m shuttle run test (5mSRT) is among the most widely used repeated sprint tests for determining a player’s match-related-fitness [3,4,5]. This test, adopted by the Welsh Rugby Union and modified by the Sports Science Institute of South Africa [4], has participants perform 6 × 30-s maximal shuttle sprints with 35-s recovery in-between. Players run the greatest possible distance for 30-s by going and returning 5 m, then 10 m, then 15 m, then 20 m, etc. Four physical parameters have been calculated during the 5mSRT: the best distance (BD), the total distance (TD), the delta distance, and the fatigue index (FI) [4]. Boddington et al. [4] demonstrated a very high reliability (Intra-class correlation coefficient (ICC) = 0.98) for TD and a moderate reliability (ICC = 0.86) for BD. However, poor reliability was reported for delta distance (ICC = 0.74) and FI (ICC = 0.74) [4].

On the other hand, total duration of the 5mSRT is about 6 min (i.e., 6 × 30-s sprints with 35-s rest in-between), which could solicit aerobic and anaerobic metabolisms; therefore, generating high levels of fatigue, muscle damage, and inflammation. Research shows performance in the 5mSRT is associated with a combination of factors including body mass, strength, and aerobic ability [6]. Moreover, the shuttle form of the 5mSRT (multiple acceleration, deceleration, and reverse-acceleration) makes it very specific to team sports such as field hockey [7], rugby [8], and soccer [9]. These characteristics of the 5mSRT make it widely used for physical performance evaluation and training purposes [5].

Physical exercise is often associated with an increase in markers of muscle damage and inflammation [10,11,12]. Interestingly, the investigation of creatinine kinase (CK) before and after physical exercise could be an important tool for coaches and clinicians [13]. Additionally, CK change from before to after physical exercise relates to the form and intensity of the exercise [13]. Furthermore, higher levels of CK accompanied by a decreased physical exercise could be an index of overtraining [14]. Taking into consideration that lactate dehydrogenase (LDH) provides information about muscle metabolism, examination of LDH responses could help coaches and physicians identify the appropriate level of training and the type of metabolic adaptation to exercise [14]. In this context, it is well known that CK, LDH, aspartate aminotransferase (ASAT), and alanine aminotransferase (ALAT) are markers of muscle and liver damages and fatigue during exercise [10]; it has been reported that these parameters are increased immediately after the Wingate test [15,16,17], repeated sprint exercise [1,18,19,20,21,22,23], the Yo-Yo intermittent recovery test [24], a training session [10], or competitions [25,26,27]. Additionally, after long-duration exercise, Hammouda et al. [24] showed increases in muscle damage markers (i.e., CK and LDH) 3 min after the level−1 Yo-Yo intermittent recovery test, confirming higher anaerobic solicitation of the test. In addition, it has been reported that a 30-s Wingate test is of sufficient intensity and duration to induce significant increases in muscle damage biomarkers (i.e., ASAT, ALAT, CK, and LDH) [16,17]. Furthermore, muscle damage biomarkers (i.e., ASAT, ALAT, CK, and LDH) are reported to increase immediately following repeated sprint ability test (5 × [6 s of maximal cycling sprint + 24 s of rest]) [20]. Similarly, it has been reported that C-reactive protein (CRP), a common marker of inflammation, increases in response to physical exercise [12].

Although the development of exercise-induced muscle damage is well documented during different high-intensity intermittent exercise protocols, including for example the 30-s Wingate test [16,17] and the 5 × 6-s of maximal cycling sprint + 24 s of rest [20], it is noteworthy that biochemical measures of muscle damage and inflammation have not yet been evaluated during the 5mSRT. Furthermore, sprint duration is a critical factor influencing the magnitude of muscle fatigue [28]. Each sprint of the 5mSRT is of 30 s [20]. During a single, short duration sprint (≤6 s), ~50% of ATP is supplied by the PCr degradation while anaerobic glycolysis and oxidative metabolism provide the rest. With increasing sprint duration, PCr depletion gradually occurs (up to 80%) during 30 s [29,30]. Consequently, a progressive greater contribution of anaerobic glycolysis associated with greater metabolic perturbation in muscle fibers (e.g., increased [Pi] and [H^+^]) could be an origin of larger muscle fatigue by alteration in excitation–contraction coupling [31]. The duration of the first repetition of the 5mSRT could be similar to the duration of a 30-s Wingate test. During the 30-s Wingate test, there is an increase in acidity within the muscle fibers due to high levels of lactic acid production; therefore, the energy production is inhibited and the work capacity limited [32].

Although the 5mSRT is widely used across a multitude of sports, the scientific basis (e.g., biochemical responses) of this test have been poorly studied. As a result, the primary purpose of the present study was to investigate the acute effect of the 5mSRT on biomarkers of muscle damage and inflammation. We hypothesized that the 5mSRT would generate significant increases in the biomarker levels.

In order to identify the optimal duration of recovery following physical exercise, previous studies examined the delayed responses of muscle damage and inflammation to different exercise protocols [1,10,11,15,33,34,35,36]. It has been reported that CK increases immediately after 30 s of high-intensity exercise and returns to baseline levels after a 24 h recovery period [15]. However, this period was not sufficient to recover CK baseline level following repeated sprint ability (RSA) exercise. Eryılmaz et al. [1] reported that at 24 h after RSA, CK remained elevated, whereas LDH returned to baseline. Similarly, Keane et al. [23] showed an elevated level of CK at 24 h, 48 h, and 72 h following RSA, with peaking at 24 h. Moreover, 48-h recovery after resistance training was not sufficient to return CK, ASAT, and LDH to resting values [10,11]. Importantly, muscle damage remained higher throughout the 72-h recovery period after a soccer [33] or a rugby [34,35] match. Discrepancies between findings have been linked to the nature, duration, and intensity of the exercise protocol utilized [10,11,36].

Therefore, considering that the 5mSRT has specific characteristics in term of intensity and duration and that the effect of such exercise protocol on muscle damage and inflammation is not yet established, the second purpose of the present study was to investigate the effects of the 5mSRT on the delayed (i.e., 72 h after) biomarkers of muscle damage and inflammation. We hypothesized that 72 h would be sufficient to recover from the 5mSRT.

While previous studies have compared different fatigue calculation formulas during repeated sprints exercise, no study has utilized the 5mSRT. In this context, Glaister et al. [37] indicated that the calculation of fatigue via the percentage decrement (PD) (i.e., taking into consideration all repetitions) is better than using the fatigue index (FI) (i.e., 1 or 2 first sprints compared to 1 or 2 last sprints). However, differences may exist between repeated sprints exercise and the 5mSRT. Indeed, the best performance could be observed during the three first sprints in the repeated sprints exercise, while it is always the first one in the 5mSRT. In addition, the previously investigated repeated sprint tests are of very short-duration (i.e., 6 to 12 repetitions of 3 to 6 s) and of shorter-duration than the 5mSRT (i.e., 6 repetitions of 30 s). In addition, Boddington et al. [4] demonstrated a poor reliability for the FI. Therefore, the third aim of this study was to test the reproducibility of the data and to examine the correlation between the calculation of fatigue using PD and FI formula and the markers of muscle damage and inflammation. Additionally, the perception of muscle soreness (DOMS), recovery (PRS), and exertion (RPE) will be examined and tested for correlation with these indices, as previous studies report significant increases in RPE scores after the 5mSRT [38,39]. We hypothesized that PD will be more representative of fatigue during the 5mSRT than FI.

## 2. Materials and Methods

### 2.1. Participants

Fifteen amateur male team (soccer (*n* = 8), rugby (*n* = 3), handball (*n* = 4) sports players (age: 20 ± 3 yrs, height: 173 ± 7 cm, body-mass: 67 ± 7 kg) provided their informed consent to voluntarily take part in the study. Participants trained at least 4 days per week for an average of 2 h per day. The study was conducted according to the Declaration of Helsinki and the protocol was fully approved by the local Research Ethics Committee (CPP: N°0098/2018). The criteria for participants’ inclusion were: non-smokers, no pathological sleep disorders (i.e., the Pittsburgh Sleep Quality Index was <5 [40]), no consumption of alcohol or foods rich in antioxidants/polyphenols (e.g., coffee, blueberries, green tea, pomegranate, cherries, grapes, curcuma, red wine, and dark chocolate), no injury, and no use of vitamins or anti-inflammatory drugs during the experimental period and for at least two months prior to study commencement.

### 2.2. Experimental Design

After a one-week familiarization session, participants were required to perform the 5mSRT at 17h00. During the period between the familiarization session and testing or even after testing exercise, they participated in only low intensity training sessions (i.e., jogging, technical, tactical). Additionally, participants ate a standardized meal before at least 4 h of the test session [41]. Furthermore, in the 2 h before the test session, subjects were asked to drink only 500 mL of water to ensure proper hydration [42]. To evaluate post-exercise fatigue, blood samples were collected from a forearm vein after 5 min of seated rest, 5 min after the 5mSRT, and 72 h after the 5mSRT for the measurement of CK, LDH, ASAT, ALAT, and CRP. Muscle soreness perception was recorded via delayed onset muscle soreness (DOMS). The DOMS consists of an 11-point scale ranging from zero (no soreness) to 10 (very, very sore) before, after the 5mSRT, and 72 h after the 5mSRT. Before, during, immediately, and 72 h after the 5mSRT, participants provided their subjective RPE score via the 11-point scale. RPE mean during the 5mSRT was calculated via the following formula:

RPE (AU) = Sum of RPE scores after all repetitions/Number of repetitions(1)

Before, after, and 72 h after the 5mSRT, each participant completed the 11-point perceived recovery scale (PRS) [43]. A passive recovery was permitted during the 72 h (after the 5mSRT).

### 2.3. 5-m Shuttle Run Test

During this test, participants performed maximal shuttle sprints for 6 × 30 s with 35-s recovery in-between. Participants maximally sprinted the greatest possible distance for 30 s by going and returning 5 m, then 10 m, then 15 m, then 20 m, etc. After each 30 s, participants were allowed 35 s of recovery, during which they returned to the starting position to prepare for the next round. This process was performed six times [4].

As calculated previously by Boddington et al. [4], the following indices were recorded:

- BD (m) = the greatest distance covered during a 30-s shuttle,
- TD (m) = total distance covered during the six 30-s shuttles,

FI (%) was calculated as follows:

FI (%) = [(((shuttle 1 + shuttle 2)/2) − ((shuttle 5 + shuttle 6)/2))/((shuttle 1 + shuttle 2)/2)] × 100(2)

Additionally, the percentage decrement (PD) during the 5mSRT was calculated as follows:

PD (%) = [((BD × number of sprints) − TD)/(BD × number of sprints)] × 100(3)

### 2.4. Blood Analyses

The collected blood samples (12 mL) were placed in an ice bath and centrifuged immediately at 2500 r/min (× g) and 4 °C for 10 min. Aliquots of the resulting plasma were stored at −80 °C for analysis. All samples were analyzed in the same run to eliminate inter-assay variance. All assays were performed in duplicate within the same laboratory with simultaneous use of a control serum from Randox. CK, LDH, ASAT, and ALAT were determined from the plasma of the heparinized tube. CRP was determined from the tube without anticoagulant. The intra-assay coefficients of variation for CK, LDH, ASAT, ALAT, and CRP were respectively 1.3%, 0.2%, 1.1%, 1.5%, and 1.16%. The 340 nm kinetics method was used to determine CK, LDH, ASAT, and ALAT. These parameters were assessed as they reflect exercise induced muscle and hepatic damage. CRP concentrations and markers of inflammation were determined using the immunoturbidimetric method.

### 2.5. Statistical Analysis

Statistical tests were performed using STATISTICA software (StatSoft, Paris, France; version 10); data are presented as mean ± standard deviation (SD). G*power software (version 3.1.9.2; Kiel University, Kiel, Germany) [44] calculated the required sample size. Values for α were set at 0.05 and power at 0.95. Based on Eryılmaz et al. [1] and discussions between the authors, effect size was estimated to be 1.03. The required sample size was twelve. After normality confirmation by the Shapiro–Wilk’s test, paired sample t-tests were used to determine whether two familiarization sessions were sufficient to eliminate any learning effects during the study. The ICC and the standard error of the measurement (SEM) were calculated for BD, TD, FI, and PD. ICC values over 0.75 were considered as excellent reproducibility, ICC values between 0.4 and 0.75 were considered as good reproducibility, and ICC values less than 0.4 was considered as poor reproducibility [45]. The Shapiro–Wilk’s test confirmed that LDH, ASAT, ALAT, and performance during the 5mSRT were normally disturbed. Performance during the 5mSRT was assessed using a one-way ANOVA (6 repetitions). LDH, ASAT, ALAT were assessed using a one-way ANOVA (3 times (before, 5 min and 72 h after the 5mSRT). When appropriate, significant differences between means were tested using the Bonferroni post hoc test. Effect sizes were calculated as partial eta-squared (ηp^2^) to estimate the meaningfulness of significant findings. ηp^2^ values of 0.01, 0.06, and 0.13 represent small, moderate, and large effect sizes, respectively.

However, when normality was not confirmed, a Friedman nonparametric analysis of variance (ANOVA) was used and the effect size was estimated by the Kendall’s coefficient of concordance. Pairwise comparisons were conducted using a Wilcoxon test on CRP, DOMS, RPE, and PRS.

Correlations between physical performance during the 5mSRT and markers of muscle damage and inflammation, as well as RPE, PRS, and DOMS, were tested using the Pearson and Spearman tests.

Significance was accepted for all analyses at the level of *p* < 0.05. Exact *p* values have been given; results given as “0.000” in the statistics output have been reported as “<0.0005”. In order to calculate the percent increase for all parameters, Δ was calculated as follows:

Δ (%) = [((Higher value − Minimum value))/(Higher value)] × 100(4)

## 3. Results

### 3.1. Reproducibility of Measurement between Test-Retest

There was no significant difference between the test and retest for BD (t = −0.91; *p* = 0.37), TD (t = −0.93; *p* = 0.36), FI (t = −0.34; *p* = 0.73), and PD (t = 0.10; *p* = 0.91). The ICC and SEM showed good reliability for BD (ICC = 0.47 and absolute SEM = 2.92), TD (ICC = 0.65 and absolute SEM = 15.81), and PD (ICC = 0.53 and absolute SEM = 0.84) and poor reliability for FI (ICC = 0.28 and absolute SEM = 1.46).

### 3.2. The 5-m Shuttle Run Test

**Figure 1:**
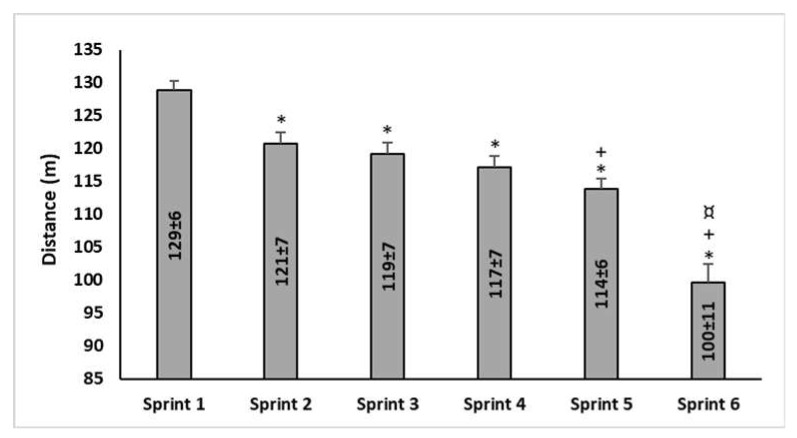
Distances covered in each 30-s sprints repetition (in meter) of the 5mSRT. *: Significantly shorter than the 1st sprint; +: Significantly shorter than the 2nd and the 3rd sprints; ¤: Significantly shorter than the 5th and the 6th sprints.

Distances covered in each repetition (in meter) during the 30-s sprints of the 5mSRT are presented in Figure 1. The one-way ANOVA revealed a significant main effect of repetitions on related performance of the 5mSRT (F(1,15) = 82.25, *p* < 0.0005, ηp^2^ = 0.85). The distance covered in each repetition from the 2nd to the 6th sprint was significantly shorter by 6.3%, 7.5%, 9.1%, 11.6%, and 22.7%, respectively than the 1st sprint (i.e., BD; *p* < 0.0005). Moreover, the distance covered in the 5th sprint was significantly shorter than the 2nd (5.6%, *p* < 0.0005) and the 3rd (4.4%, *p* = 0.01) sprints. Finally, the distance performed in the last sprint was significantly shorter (*p* < 0.0005) than the 1st (22.7%), 2nd (17.5%), 3rd (16.4%), 4th (14.8%), and 5th (12.5%) sprints.

### 3.3. Biochemical Parameters

**Figure 2:**
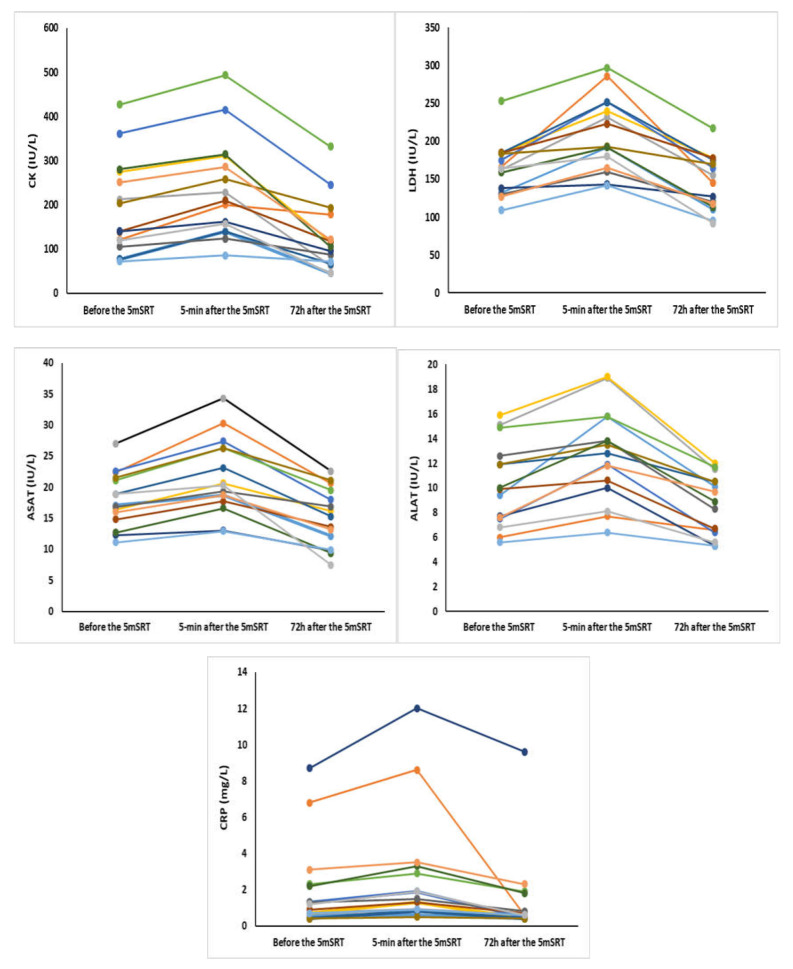
Biochemical parameters recorded before, 5 min and 72 h after the 5mSRT for all participants.

Table 1 reports the mean values for CK, LDH, ASAT, ALAT, and CRP before, 5 min and 72 h after the 5mSRT, as well as *p* value and effect sizes, and Figure 2 represents individual changes of these parameters recorded before and 5 min and 72 h after the 5mSRT. Results showed that all biochemical concentrations were increased 5 min after compared to before the 5mSRT. Furthermore, all biochemical concentrations were decreased 72 h after compared to before (*p* < 0.05) and 5 min after the 5mSRT (*p* < 0.001).

### 3.4. Delayed Onset Muscle Soreness

There was a significant main time effect on DOMS (test = 26.77, *p* < 0.0005, Kendall’s W = 0.89). Statistical analysis showed that DOMS scores were 65.1% and 15% higher before compared to 5 min (*p* = 0.0009) and 72 h (*p* = 0.01) after the 5mSRT (Table 2). In addition, DOMS scores were 71.4% higher 72 h compared to 5 min after the 5mSRT (*p* = 0.0006) (Table 2).

### 3.5. Rating of Perceived Exertion Scale

There was a significant main time effect on RPE (test = 26.81, *p* < 0.0005, Kendall’s W = 0.89). Statistical analysis showed that mean RPE and end RPE scores were 62.4% and 74.4% higher compared to before (*p* = 0.0006) and were 72.7% and 81.3% higher compared to 72 h after (*p* = 0.0006) the 5mSRT respectively (Table 2). In addition, RPE scores were 22.2% higher before compared to 72 h after the 5mSRT (*p* = 0.03) (Table 2).

### 3.6. Perceived Recovery Status Scale

There was a significant main time effect on PRS (test = 27.84, *p* < 0.0005, Kendall’s W = 0.92). Statistical analysis showed that PRS scores were 50.2% and 6.5% higher before compared to 5 min (*p* = 0.006) and 72 h (*p* = 0.01) after the 5mSRT (Table 2). In addition, PRS scores were 53.6% higher 5 min compared to 72 h after the 5mSRT (*p* = 0.0006).

### 3.7. Correlations

As presented in Table 3, the statistical analysis showed significant correlations between physical parameter (PD, FI, TD, and BD) values and markers of muscle damage and inflammation, as well as RPE, PRS, and DOMS.

## 4. Discussion

The purposes of the present study were (i) to investigate the effects of biochemical (i.e., CK, LDH, ASAT, ALAT, and CRP) and perceptual (i.e., DOMS, RPE, and PRS) responses to the 5mSRT and (ii) to examine the correlations between the physical performance parameters during the 5mSRT and markers of muscle damage and inflammation as well as RPE, PRS, and DOMS.

Findings indicate the 5mSRT resulted in significant increases in muscle damage and inflammation. Specifically, CK, LDH, ASAT, and ALAT were identified as markers of muscle damage and fatigue during exercise [10,11]. The significant increases in CK, LDH, ASAT, and ALAT immediately after the 5mSRT support evidence of exercise induced muscle damage. The present results are in agreement with other studies reporting increases in these parameters following physical exercise, such as repeated sprints [1,18,19,20,21,22,23] or following a training session [10]. In the present study, the shuttle form (i.e., multiple acceleration, deceleration, and reverse-acceleration) appeared to result in muscle damage. According to our study, other forms of exercise (i.e., repeated sprinting together with 10-m rapid deceleration at the end of each sprint or 15 × 30-m sprints interspersed by 60 s of rest) generate increased muscle damage [1,19,22]. Furthermore, decreases in physical performance during the 5mSRT and increases in markers of muscle damage could be explained by the frequent abnormalities of contractile material and the cytoplasmic organelles at the ultrastructural level which may result from eccentric muscle contractions during the landing phase of sprinting [1]. In this context, the current study revealed that DOMS scores increased by 65.1% in response to the 5mSRT, which may help explain the significant reductions in distance recorded from the 1^st^ sprint. Additionally, the inability to reproduce the same sprint performance during the 5mSRT could be related to the short recovery between repetitions (i.e., 35 s), thus limiting re-synthesis of phosphocreatine stores [2], which are a consequence of a high level of anaerobic lactic acid production [32]. More importantly, the 5mSRT is a maximal physical exercise, and thus could generate a temporary rising of hepatic and muscle enzymes [17]. The higher levels of CK, LDH, ASAT, and ALAT recorded after the 5mSRT could be a consequence of higher exercise intensity. The higher intensity of the 5mSRT could be supported by increased RPE and lowered PRS responses. RPE scores increased by 62.4% during and by 74.4% at the end compared to before the 5mSRT. It has been reported that changes in muscle damage depend on exercise intensity [46]. In support of this idea, Grazioli et al. [47] showed moderate volume of sprint bouts, with or without change of direction, does not induce increases in CK. However, Kingsley et al. [48] reported increased CK concentrations after an intermittent session of shuttle run training at different intensities in amateur soccer players. Deminice et al. [49] showed increased levels of CK following a set of eight maximal swims along 100 m with 10 min recovery in-between. In this study, CK and LDH increased by 21.3% and 21%, respectively after the 5mSRT. The higher levels of these parameters after the 5mSRT could be related to its metabolic and mechanical demands [13]. In fact, increases in CK and LDH levels after the 5mSRT could be clarified by a disturbance in the homeostasis of internal free calcium ions and intramuscular contractile functions [15]. Additionally, the higher levels of CK and LDH could be related to the local tissue damage with sarcomeric degeneration from Z-disk fragmentation [13]. CK is indicative of muscle necrosis [13], and it has been reported that CK and LDH flee into the interstitial fluid and are taken up via lymphatic vessels and returned to circulation [17]. ASAT is included in striated muscle and augmented with ALAT and CK after physical exercise [17]. Thus, the simultaneous change of CK, LDH, ASAT, and ALAT in the present study is greatly affected by muscular activity, as opposed to hepatic injury [17]. In fact, the higher levels of ALAT are indicative of muscle injury in the absence of liver disease, which is consistent with the higher levels of CK and LDH after the 5mSRT. Moreover, muscle damage induced by the 5mSRT is indicative of inflammatory responses, which is confirmed in this study by the elevation of CRP [23,50,51,52].

Importantly, present results showed 72 h was sufficient to recover from fatigue induced by the 5mSRT. Furthermore, levels of muscle damage (i.e., CK, LDH, ASAT, and ALAT) and inflammatory biomarkers (i.e., CRP) returned to baseline 72 h after the 5mSRT. The present results are in agreement with previous studies reporting faster recovery of pre-exercise muscle damage within the 72 h following exercise [15,53,54]. In this context, 24-h rest was sufficient to recover from 30 s of high-intensity exercise [15], 48-h rest was sufficient to recover from one week of high-intensity training [54], and 72-h rest was sufficient to recover from 90 min of intermittent shuttle running and walking [53]. However, our results are in contrast with others reporting that muscle damages remain elevated three to seven days following anaerobic and/or aerobic based exercise [19,22,23,51,55]. It has been shown that CK remains elevated above baseline at 72 h following RSA test (15 × 30-m sprints with a 10-m deceleration) interspersed by 60 s [19] or 65 s of rest [23], and CK and LDH remained elevated at 72 h following acute plyometric exercise [51], weightlifting exercise session [10,11], and soccer [33] or rugby [34,35] matches. These contradictions confirm that time course of recovery following exercise-induced muscle damage depends on the exercise protocol utilized [36].

In fact, weightlifting sessions (90 min) [10,11] and soccer or rugby matches [33,34,35] are characterized by the involvement of multiple muscle groups, higher range of motion (e.g., during jumping), and longer duration than the 5mSRT, which could delay muscle damage. In order, to attenuate the muscle damage responses in such exercise types, it was suggested that the more solicited muscle groups and joints should be trained at similar intensities by using the same muscle movements as the destructive exercises [22].

Besides the exercise protocol, it has been also suggested that participant training level may play a crucial role in determining the recovery kinetic of muscle damage, with faster recovery showed in athletic population [13,14]. The present results confirm this suggestion showing that, when using a trained population, the levels of muscle damage (i.e., CK, LDH, ASAT, and ALAT) and inflammatory biomarkers (i.e., CRP) were lower at 72 h following the 5mSRT compared to the baseline values (i.e., before the 5mSRT). These results indicate that a fast-full recovery from the 5mSRT, inducing muscle damage and inflammation, has been already reached before 72 h. This highlights the necessity to conduct further studies with more post-exercise time point sampling to identify the optimal recovery period (i.e., shortest period that return values to baseline [10]). The present fast recovery kinetics may be due to training adaptations. It was previously shown that training for a long period can induce activation of the redox sensitive transcription factors such as NF-kB, thus activating the production of endogenous antioxidants and reinforcing the body against muscle and oxidative damages [56,57].

Contrary wise, based on correlational analysis, in contradiction with our second hypothesis and the results of Glaister et al. [37,58], the results of the present study reported significant correlations between (*i*) increases in the FI and PD and (*ii*) the increases in pre-/post-5mSRT percentage changes in muscle damage and inflammation markers as well as RPE, PRS, and DOMS. This supports the notion that both FI and PD calculations reflected the 5mSRT induced fatigue. However, the ICC and SEM analysis showed a good reliability for PD (ICC = 0.53 and absolute SEM = 0.84) and a poor reliability for FI (ICC = 0.28 and absolute SEM = 1.46). This confirms our second hypothesis and supports the data of Glaister et al. [37,58]. In the same context, Boddington et al. [4] demonstrated a poor reliability for FI during the 5mSRT.

While we are confident in the results, this study is not without limitations. First, plasma volume may change as a result of the 5mSRT; however, in the present study, biochemical concentrations were not corrected for plasma volume changes. Future studies should account for changes in plasma volume when calculating biochemical concentrations. Second, the lack of a control group was another limitation in order to examine the effect of training status on the muscle damage and inflammation responses during the 5mSRT. Indeed, players involved in this study have different levels of fitness, which may have influenced results. Future studies should include players with homogenous level of fitness. Lastly, we did not investigate the delayed responses (i.e., 24 and 48 h) of muscle damage and inflammation biomarkers as well as perceptual responses of fatigue, recovery, and muscle soreness following the 5mSRT. Thus, further studies should test the acute and delayed (i.e., 24, 48, and 72 h) responses of muscle damage and inflammation biomarkers as well as perceptual responses of fatigue, recovery, and muscle soreness following the 5mSRT.

## 5. Conclusions

The present study reported the 5mSRT increased muscle damage (i.e., CK, LDH, ASAT, and ALAT) and inflammation (i.e., CRP) biomarkers, as well as DOMS and RPE and reduced the PRS. Furthermore, 72 h was sufficient for recovery after the 5mSRT. Based on the results, both FI and PD calculations could be used during the 5mSRT to represent fatigue. However, the reliability of the results was stronger for PD (ICC = 0.53) than for FI (ICC = 0.28). This supports the better utilization of PD during the 5mSRT to calculate the performance reduction. Additionally, this test could be utilized during the periods of overreaching when coaches need the increases of fatigue and training load to prepare for a tapering period.

## Figures and Tables

**Table 1:** Values (mean ± standard deviation) of biochemical parameters before, 5 min and 72 h after the 5mSRT.

Parameters	before	5 Min after	72 h after	% of Increase between after and before	% of Decrease between 5 Min after and 72 h after the 5mSRT	% of Decrease between before and 72 h after the 5mSRT	ANOVA	*p* Value	Effect Size
CK (IU/L)	190.6 ± 109.1	234.6 ± 113.7 #	125.3 ± 80.5 #¤	21.3%	−46.1%	−29.3%	Test = 28.13	<0.0005	0.93
LDH (IU/L)	163.6 ± 35.1	209.9 ± 50.8 #	143.9 ± 36.6 #¤	21.0%	−30.9%	−12.2%	F = 47.13	<0.0005	0.77
ASAT (IU/L)	18.0 ± 4.4	21.7 ± 6.2 #	15.0 ± 4.7 #¤	16.2%	−30.4%	−16.4%	F = 44.46	<0.0005	0.76
ALAT (IU/L)	10.2 ± 3.4	12.7 ± 3.8 #	8.6 ± 2.4 #¤	19.4%	−30.9%	−13.0%	F = 36.09	<0.0005	0.72
CRP (mg/L)	2.1 ± 2.5	2.8 ± 3.3 #	1.4 ± 2.3 #¤	25.8%	−46.8%	−27.9%	Test = 26.77	<0.0005	0.89

#: Significant difference in comparison with before the 5mSRT. ¤: Significant difference in comparison with 5 min after the 5mSRT.

**Table 2:** Delayed onset muscle soreness (DOMS), rating of perceived exertion (RPE), and perceived recovery status (PRS) scores recorded before, 5 min and 72 h after the 5mSRT.

Parameters	Before the 5mSRT	5 min after the 5mSRT	72 h after the 5mSRT
DOMS (AU)	2.4 ± 1.0	6.7 ± 1.1 #	1.9 ± 0.7 #¤
End RPE (AU)	2.1 ± 0.6	8.1 ± 0.6 #	1.5 ± 0.6 #¤
Mean RPE(AU)	2.1 ± 0.6	5.6 ± 0.6 #	1.5 ± 0.6 #¤
PRS (AU)	6 ± 1	3 ± 1 #	7 ± 1 #¤

#: Significant difference in comparison with before the 5mSRT. ¤: Significant difference in comparison with 5 min after the 5mSRT.

**Table 3:** Correlations between the physical performance and markers of muscle damage and inflammation as well as the perception of exertion (RPE), recovery (PRS), and muscle soreness (DOMS).

		**Acute Responses**										
		**FI (%)**	**TD (m)**	**BD (m)**	**ASAT (%)**	**ALAT (%)**	**CK (%)**	**LDH (%)**	**CRP (%)**	**DOMS (%)**	**PRS (%)**	**RPE (%)**
**PD (%)**	**r**	0.92	0.82	0.78	0.74	0.73	0.71	0.73	1	0.84	0.87	0.76
	***p***	*p* < 0.0005	*p* < 0.0005	*p* = 0.001	*p* = 0.002	*p* = 0.002	*p* = 0.003	*p* = 0.002	*p* < 0.0005	*p* < 0.0005	*p* < 0.0005	*p* = 0.001
**FI (%)**	**r**	-	0.77	0.77	0.67	0.73	0.78	0.69	0.93	0.85	0.86	0.86
	***p***	-	*p* = 0.001	*p* = 0.001	*p* = 0.006	*p* = 0.002	*p* = 0.001	*p* = 0.004	*p* < 0.0005	*p* < 0.0005	*p* < 0.0005	*p* < 0.0005
**TD (m)**	**r**	-	-	0.95	0.94	0.97	0.91	0.95	0.90	0.97	0.87	0.78
	***p***	-	-	*p* < 0.0005	*p* < 0.0005	*p* < 0.0005	*p* < 0.0005	*p* < 0.0005	*p* < 0.0005	*p* < 0.0005	*p* < 0.0005	*p* = 0.001
**BD (m)**	**r**	-	-	-	0.96	0.95	0.85	0.97	0.88	0.92	0.91	0.81
	***p***	-	-	-	*p* < 0.0005	*p* < 0.0005	*p* < 0.0005	*p* < 0.0005	*p* < 0.0005	*p* < 0.0005	*p* < 0.0005	*p* < 0.0005
		**Delayed Responses**										
		**FI (%)**	**TD (m)**	**BD (m)**	**ASAT (%)**	**ALAT (%)**	**CK (%)**	**LDH (%)**	**CRP (%)**	**DOMS (%)**	**PRS (%)**	**RPE (%)**
**PD (%)**	**r**	-	-	-	0.67	0.57	0.96	0.64	0.75	0.90	0.69	0.61
	***p***	-	-	-	*p* = 0.005	*p* = 0.02	*p* < 0.0005	*p* = 0.009	*p* = 0.001	*p* < 0.0005	*p* = 0.004	*p* = 0.01
**FI (%)**	**r**	-	-	-	0.71	0.57	0.81	0.64	0.58	0.61	0.64	0.66
	***p***	-	-	-	*p* = 0.003	*p* = 0.02	*p* < 0.0005	*p* = 0.009	*p* = 0.02	*p* = 0.01	*p* = 0.009	*p* = 0.007
**TD (m)**	**r**	-	-	-	0.65	0.63	0.97	0.67	0.61	0.62	0.57	0.70
	***p***	-	-	-	*p* = 0.008	*p* = 0.01	*p* < 0.0005	*p* = 0.006	*p* = 0.01	*p* = 0.01	*p* = 0.02	*p* = 0.003
**BD (m)**	**r**	-	-	-	0.74	0.66	0.90	0.85	0.71	0.67	0.63	0.70
	***p***	-	-	-	*p* = 0.001	*p* = 0.007	*p* < 0.0005	*p* < 0.0005	*p* = 0.003	*p* = 0.006	*p* = 0.01	*p* = 0.003
